# Synthesis of NiO/Nitrogen-Doped Carbon Nanowire Composite with Multi-Layered Network Structure and Its Enhanced Electrochemical Performance for Supercapacitor Application

**DOI:** 10.3390/ma15207358

**Published:** 2022-10-20

**Authors:** Zhuanzhuan Shi, Xiaofen Li, Xiaohai Wang, Zhikai Wang, Xiaoshuai Wu

**Affiliations:** Institute of Materials Science & Devices, School of Materials Science and Engineering, Suzhou University of Science and Technology, Suzhou 215009, China

**Keywords:** multi-layered structure, NiO nanowires, nitrogen doping carbon backbone, supercapacitors

## Abstract

Multi-layered NiO nanowires linked with a nitrogen-doped carbon backbone grown directly on flexible carbon cloth (NiO/NCBN/CC) was successfully fabricated with a facile synthetic strategy. The NiO/NCBN/CC was further used as a binding-free electrode for flexible energy storage devices, showing a boosted performance including a high capacitance of 1039.4 F g^−1^ at 1 A g^−1^ and an 83.4% capacitance retention ratio. More importantly, after 1500 cycles, the capacitance retention can achieve 72.5% at a current density of 20 A g^−1^. The excellent electrochemical properties of the as-prepared NiO/NCBN/CC are not only attributed to the multi-layered structure that can help to tender unimpeded channels and accommodate the electrolyte ions around the electrode interface during the charge–discharge process, but is also due to the link between the NiO and N-doped carbon backbone and the nitrogen doping on the carbon substrate, which results in extra defects on the surface that could boost the interfacial electron transfer rate of the electrode.

## 1. Introduction

Due to the impressive advantages of supercapacitors (SCs), such as fast charge/discharge rates, long cycling, and an instantaneous stability at a higher power density, they have attracted scientific interest [1,2,3]. Among various SCs, the flexible supercapacitors have become one of the most intensely scrutinized forms of research in modern society due to the growing requests for wearable electronics. However, their practical applications are limited by the relatively low energy density in many vital fields. Generally, the composition and nanostructure of electrode materials greatly affect the performance of the flexible supercapacitors. Therefore, to meet the urgent needs of all-in-one portable and wearable electronic devices, thin and lightweight SCs with various nanostructures have been developed [4,5,6,7,8].

Typically, carbon materials, conducting polymers (Polyaniline: PANI, Poly (3,4-ethylene dioxythiophene): PEDOT), and transition metal oxides (NiO, Fe_2_O_3_) have been used as electrodes for supercapacitors. Among these materials, carbon-based materials are usually used for electrochemical double-layer capacitors (EDLCs), while conducting polymers and transition-metal oxides are often employed as electrode materials for pseudocapacitors [9,10,11]. The relatively low power density is the disadvantage of transition metal oxide-based SCs due to the poor conductivity of the electrode material. At the same time, this type of capacitor can deliver a higher energy density. In contrast, SCs using carbon materials generally provide a higher power density, but their shortcomings are a lower energy density and capacitance [12]. Furthermore, it is crucial to design the microstructure and morphology of the material to achieve a combined improvement in the performance and electrochemical properties of SCs, such as energy density, electrical conductivity, power density, specific capacitance, and so on. In this case, an approach to promoting transition metal oxides’ linkage with carbon material that are grown directly on flexible substrate could be utilized to develop the electrode architecture for flexible SCs.

As a promising material for application in SCs, transition metal oxides have captured widespread attention due to their fast faradaic redox reactions [13,14]. For example, NiO has been considered a potential electroactive material for SCs on account of its prospects of a high theoretical specific capacitance, controllable nanostructures, well-defined redox activity, and low cost [15,16]. However, the relatively poor electrical conductivity of NiO may lead to comparatively poor rate capability and low specific capacitance [3]. To address the above limitations, various forms of NiO, such as honeycomb-like NiO [17] and NiO nanobelts [18], have been designed and have demonstrated their excellent electrochemical performance. The hierarchical structure of NiO could promote the connection with the electrolyte and further facilitate ion diffusion [19]. Moreover, nanostructured NiO benefits the rate capability and specific capacitance [20,21,22]. Herein, multi-layered NiO nanowires linked with a nitrogen-doped carbon backbone that was grown directly on flexible carbon cloth (NiO/NCBN/CC) were successfully synthesized by directly depositing NiO nanowires on the outer surface and loading them into the pore of the nitrogen-doped carbon backbone (NCBN/CC) to form a multi-layered network structure. The NCBN can facilitate the growth of NiO nanowires and enhance the link between NiO and carbon cloth. The homogeneously distributed NiO nanowires and multi-layer network structure are beneficial for shortening the diffusion distances of electrons and ions. Furthermore, the nitrogen doping on the interface can improve the reaction activity and conductivity. Consequently, the NiO/NCBN/CC with a multi-layered network structure can exhibit an outstanding rate capability and a high specific capacity, which signify the potential of NiO/NCBN/CC towards supercapacitor applications.

## 2. Materials & Methods

### 2.1. Preparation of NiO NWs/CC and NiO/NCBN/CC

The NCBN/CC was synthesized according to our recent work [23]. Nickel chloride, urea, and hexadecyl trimethyl ammonium bromide were analytically pure and purchased from Sigma-Aldrich (Shanghai, China). NiO nanowires were grown in situ on NCBN/CC to prepare a NiO/NCBN/CC electrode. Briefly, nickel chloride (0.03 mol L^−1^) and urea (0.045 mol L^−1^) were dissolved in ultrapure water under vigorous stirring; then, hexadecyl trimethyl ammonium bromide (0.015 mol L^−1^) was added to the homogeneous solution to control the morphology of NiO. A piece of NCBN/CC or carbon cloth was subsequently soaked in the above solution (while keeping the electrode stand upright in the solution), followed by heating at 150 °C for 6 h. Finally, the as-synthesized electrode was taken out until it cooled to room temperature and was then washed several times and calcinated at 350 °C for 2 h. The electrodes are denoted as NiO NWs/CC and NiO/NCBN/CC. The electronic microbalance was used to determine the mass of NiO/NCBN/CC electrode. Field emitted scanning electron microscope (FESEM, JEOL, JSM-7800F, Japan) was used to characterize the electrode morphology. X-ray photoelectron spectroscopy (XPS) and X-ray photoelectron spectrometry were performed (Thermo Fisher Scientific Inc., ESCALAB 250Xi, Waltham, MA, USA).

### 2.2. Electrochemical Measurements

A CHI 660E electrochemical workstation was used to test the electrochemical performances. The three-electrode cell used a platinum sheet, a saturated calomel electrode, and the as-prepared electrode; the electrolyte was used 6 mol L^−1^ of KOH solution. The electrochemical performances were analyzed by cyclic voltammetry (CV), galvanostatic charge–discharge (GCD), and electrochemical impedance spectroscopy (EIS). For the three-electrode cell, the potential window of CV and GCD curves was 0–0.5 V and 0–0.35 V, respectively. The charge–discharge curve was calculated as follows:

$$Cs=\frac{I∆t}{m∆V}$$

where *I* represents the discharge current (A), ∆*t* denotes the discharge time (s), ∆*V* corresponds to the potential window (V), m stands for the mass of active materials (g), and *Cs* refers to specific capacitance (F g^−1^).

## 3. Results and Discussion

**Figure 1:**
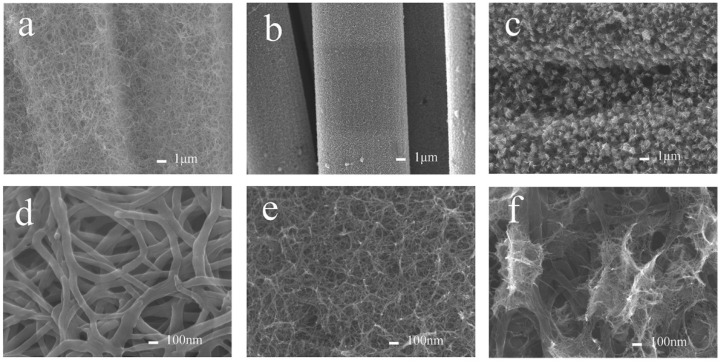
FESEM image of NCBN/CC (**a**,**d**), NiO NWs/CC (**b**,**e**), and NiO/NCBN/CC (**c**,**f**).

The detailed morphological and structural characterization of NCBN/CC, NiO NWs/CC, and NiO/NCBN/CC were examined by FESEM at various magnifications. As shown in Figure 1a,d, it is evident that a uniform distribution of randomly oriented nitrogen-doped carbonized nanowires forms a wire-like structure; the individual nanowires are approximately 60–100 nm in width. When NiO nanowires are directly grown on carbon cloth fibers (Figure 1b,e), the structure is denser, while the diameter of the NiO nanowires is much thinner than carbon nanowires. After the NiO nanowires were deposited on NCBN/CC, the multi-layered network structure of NiO/NCBN/CC could be observed (Figure 1c,f) and the diameter of the NiO nanowire was much smaller than nitrogen-doped carbonized nanowires; the EDS mapping (Figure S1) indicates the uniform distribution of elements evenly across whole electrode material. In addition, the NiO nanowires intercrossed with each other in the in situ-grown NCBN/CC and the pores of the nitrogen-doped carbon backbone, and many of the connection points between the NiO nanowire and NCNWs also can be observed in Figure 1e. Such a multi-layered network structure could improve the contact area for the electrolyte on the electrode interface, and the space among the nanowires not only promoted ion diffusion and charge transport [14,24] but also served as a low-resistance pathway for ion migration during electrochemical reactions [25].

**Figure 2:**
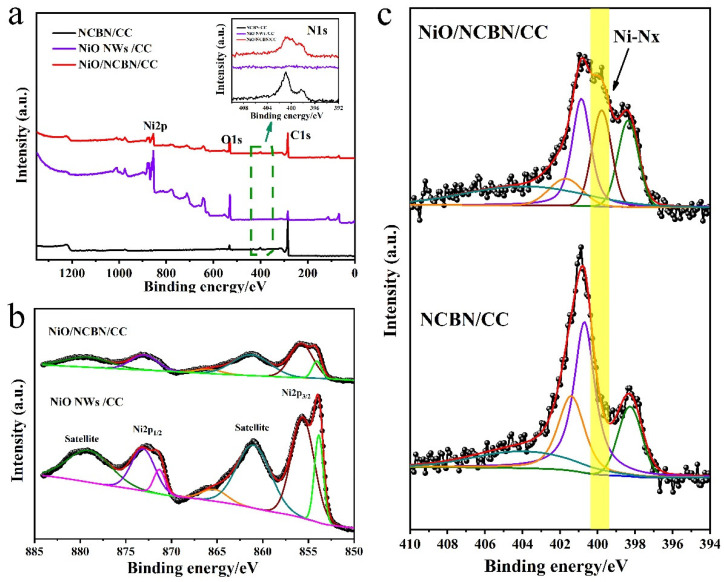
(**a**) XPS survey spectra of NCBN/CC, NiO NWs/CC, and NiO/NCBN/CC; (**b**) Ni 2p spectrum of NiO/NCBN/CC and NiO NWs/CC; (**c**) N1s spectrum of NCBN/CC and NiO/NCBN/CC.

The chemical bonding states were used to identify the formation of links between the NiO and N-doped carbon backbone. As shown in Figure 2a, the signals of C 1s, O 2p, and N1s around 285 eV, 530 eV, and 401 eV in the broad scan survey spectra of NCBN/CC demonstrate that the composites comprise C, O, and N elements. After the NiO nanowires grew on the surface of the NCBN/CC, a new peak located at 850 eV~885 eV was observed. The C 1s spectrum (Figure S2a) of NiO/NCBN/CC exhibits three peaks for the C–C/C=C, C–N/C–O, and O–C=O/C=O bands at 284.6, 285.6, and 288.9 eV, respectively [26]. Moreover, the O 1s spectrum (Figure S2b–d) displays three deconvoluted peaks for Ni–O, C=O, and C–O–C bonds at 529.6, 531.4, and 533.4 eV [27]. For the Ni 2p spectra in Figure 2b, the peaks’ values were 854.74 eV and 871.58 eV, exactly matching NiO, which corresponds to Ni 2p_3/2_ and Ni 2p_1/2_, respectively, and together with the two satellite peaks at the high energy side [28]. The link between the NiO and N-doped carbon backbone could be confirmed by the N 1s spectrum shown in Figure 2c; the N1s peak of the N-doped carbon backbone was well deconvoluted into four peaks located at 398.57 eV, 400.73 eV, 401.1 eV, and 403.44 eV attributed to pyridine N (N-6), pyridine or pyrrole N (N-5), quaternary N (N-Q), and oxidized N (N-X), respectively [29]. Interestingly, a new peak located at 399.8 ± 0.1 eV could be observed after the NiO nanowires had grown on the surface of NCBN/CC, which corresponds to the bond of Ni-Nx [30,31]. All the results above confirm the incorporation of the NiO nanowires into the N-doped carbon backbone network.

**Figure 3:**
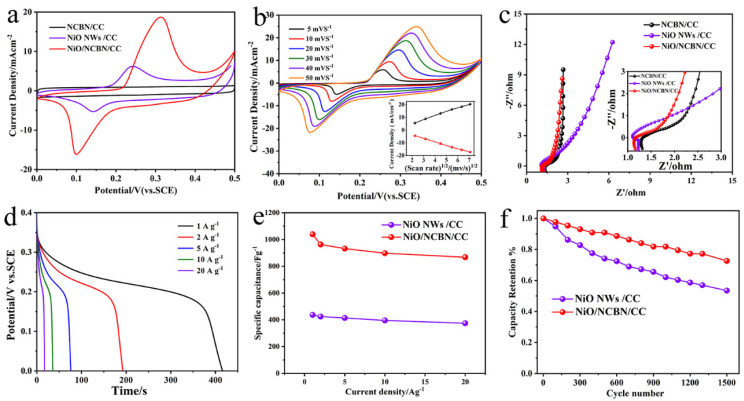
(**a**) CV curves of NCBN/CC, NiO NWs/CC, and NiO/NCBN/CC (30 mV s^−1^); (**b**) typical CV curves of NiO/NCBN/CC electrode at different scanning rates (5 mV s^−1^ to 50 mV s^−1^, insert: scan rate vs. redox peak current); (**c**) EIS of NCBN/CC, NiO NWs/CC and NiO/NCBN/CC; (**d**) GCD curves of NiO/NCBN/CC; (**e**) specific capacity of NiO/NCBN/CC and NiO NWs/CC; (**f**) cycling performances during 1500 cycles at 10 Ag^−1^.

The electrochemical properties of the different electrodes were characterized using CV and GCD measurements in a 6 mol L^−1^ KOH aqueous solution. The CV curves of NCBN/CC, NiO/NCBN/CC, and NiO NWs/CC within a potential window of 0–0.5 V are shown in Figure 3a. The current density of NiO/NCBN/CC is much higher than NCBN/CC and NiO NWs/CC, which indicates that the NiO/NCBN/CC electrode delivers a much larger charge storage ability. When the scan rate was increased (Figure 3b), the oxidation peaks and reduction peaks shifted to a more positive position and more negative position, respectively. Furthermore, the CV curves of the NiO/NCBN/CC electrode display a symmetrical shape (Insert of Figure 3b), which suggests a decrease in polarization loss and an increase in coulombic efficiency. From the Nyquist plots shown in Figure 3c, the internal resistance (*Rs*) and charge transfer resistance (*Rct*) of the NiO/NCBN/CC electrode are estimated to be 1.15 Ω and 0.6 Ω, while those of NCBN/CC are 1.3 Ω and 1.2 Ω, respectively. The result shows that the NiO/NCBN/CC electrode exhibits a smaller *Rs* and *Rct* than the behavior of NCBN/CC and NiO NWs/CC, demonstrating that the elaborate architecture of the multi-layered NiO/N-doped carbon backbone network remarkably improves the electroconductivity and electroactive surface area. The galvanostatic discharge curves of the multi-layered network structure at different current densities are conducted in a potential range of 0–0.35 V, and the non-linearity of the discharge curves of NiO/NCBN/CC (Figure 3d) shows the pseudocapacitance behavior arising because of the redox reaction at the electrode and electrolyte interface. In addition, due to the relative increase the in kinetic irreversibility of the OH^-^ ions to the NiO surface at higher current densities, the discharging curves for all current densities are not symmetrical [32]. The galvanostatic discharge curves of the different electrodes show that the NiO/NCBN/CC electrode possesses the longest discharging time (Figure S3), demonstrating the improvement of the specific capacitance of the NiO/NCBN/CC electrode.

The rate capabilities and specific capacitance of NiO/NCBN/CC and NiO NWs/CC at various current densities are shown in Figure 3e. For the NiO/NCBN/CC electrode, the specific capacitance is up to 1039.4 F g^−1^ at 1 A g^−1^, which is 2.4-fold higher than the NiO NWs/CC electrode (436.4 F g^−1^). The corresponding specific capacitances are 963.2 F g^−1^, 932.4 F g^−1^, 898 F g^−1^, and 867.6 F g^−1^ at 2 A g^−1^, 5 A g^−1^, 10 A g^−1^, and 20 A g^−1^, and the capacitance retained ratios are 92.7%, 89.7%, 86.4%, and 83.4%, respectively. By comparison, the specific capacitance of the porous NiO/NCBN/CC is significantly better than that of NiO nanomaterials with different nanostructures reported in recent years, as shown in Table S1. When the current density increased from 1 to 20 A g^−1^, 83.4% of the original capacitance of the NiO/NCBN/CC electrode was retained, which suggests a better rate capability and a potential benefit from their multi-layered network structure. In addition, the cycle reliability of NiO/NCBN/CC and NiO NWs/CC electrodes shown in Figure 3f indicates that the capacitance retention of NiO NWs/CC is only 53.4% after 1500 cycles at a high current density of 20 A g^−1^, while a 72.5% capacitance retention for NiO/NCBN/CC was obtained, which reveals the remarkable cycle performance of NiO/NCBN/CC electrode.

**Figure 4:**
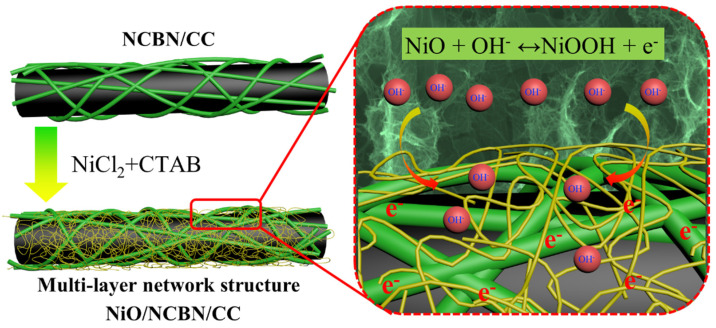
Schematic illustration of the mechanism involved in the high performance of NiO/N-doped carbon nanowires with multi-layered network structure.

The electrochemical performances of the as-prepared NiO/NCBN/CC are believed to benefit from the interconnection between the NiO and N-doped carbon backbone and the advantages of a multi-layered network structure based on the above results (Figure 4). On the one hand, the multi-layered network structure of NiO/NCBN/CC can help to tender unimpeded channels and accommodate a large number of electrolyte ions, which can effectively reduce the charge transfer resistance during the rapid charge and discharge process and promote a fast Faraday reaction to achieve a high rate capability. In addition, the 3D morphology could serve as an effective conduction path for electron transmission. Furthermore, the nanoscales and homogeneous distribution of the NiO nanowires and N-doped carbon nanowires can offer enough electroactive sites for rapid redox reactions. Finally, the doping of heteroatoms on the carbon substrate results in extra defects on the surface, which would enhance the electrochemical performance of the materials and promote its application in supercapacitor-related materials.

## 4. Conclusions

In conclusion, a multi-layered NiO/N-doped carbon nanowire network was successfully fabricated via a facile synthetic strategy, which directly deposited NiO nanowires on the outer surface and loaded them into the pore of N-Doped carbon nanowires to form a multi-layered network structure. Benefiting from such a 3D interconnected porous architecture and the homogeneously decorated NiO and N-doped carbon backbone, the NiO/NCBN/CC shows a boosted performance, including a high capacitance of 1039.4 F g^−1^ at 1 A g^−1^ and an 83.4% capacitance retention ratio. More importantly, after 1500 cycles at a current density of 20 A g^−1^, the capacitance retention can achieve 72.5%. The as-prepared NiO/NCBN/CC display an excellent electrochemical performance that is attributed to the multi-layered structure that helps to tender unimpeded channels during the charge–discharge process, as well as the link between the NiO and N-doped carbon backbone and the nitrogen doping on the carbon substrate, which results in extra defects on the surface that can boost the interfacial electron transfer rate of the electrode.

## Supplementary Materials

The following supporting information can be downloaded at: https://www.mdpi.com/article/10.3390/ma15207358/s1. Figure S1. EDS mapping images of NiO@NCNWs-CC. Figure S2. (a) C1s spectrum of NiO@NCNWs-CC; (b–d) O1s spectrum of NiO@NCNWs-CC, NCNWs-CC and NiO-CC. Figure S3. The Galvanostatic discharge curves of NiO@NCNWs-CC, NCNWs-CC and NiO-CC. Table S1. The specific capacitance of NiO-based electrode materials. References [33,34,35,36,37,38,39,40,41,42,43] are cited in the Supplementary Materials.
