# Supplementary material for: Synthesis of NiO/Nitrogen-Doped Carbon Nanowire Composite with Multi-Layered Network Structure and Its Enhanced Electrochemical Performance for Supercapacitor Application
Source: Materials (Basel). 2022 Oct 20;15(20):7358. doi: 10.3390/ma15207358 (PMC9607312; doi:10.3390/ma15207358)
Supplement: Supplementary file 1 [file materials-15-07358-s001.zip › materials-1766093-supplementary.pdf]

Supplementary Information

Synthesis of NiO/Nitrogen-Doped Carbon Nanowire Composite with Multi-Layered Network Structure and its Enhanced Electrochemical Performance for Supercapacitor Application

Zhuanzhuan Shi, Xiaofen Li, Xiaohai Wang, Zhikai Wang and Xiaoshuai Wu *

Institute of Materials Science & Devices, School of Materials Science and Engineering, Suzhou University of Science and Technology, Su Zhou 215009, China; shizz@usts.edu.cn (Z.S.); lixiaofen586@163.com (X.L.); wangxiaohai0316@163.com (X.W.); jasonwang01033@163.com (Z.W.)

* Correspondence: wuxiaoshuai365@163.com

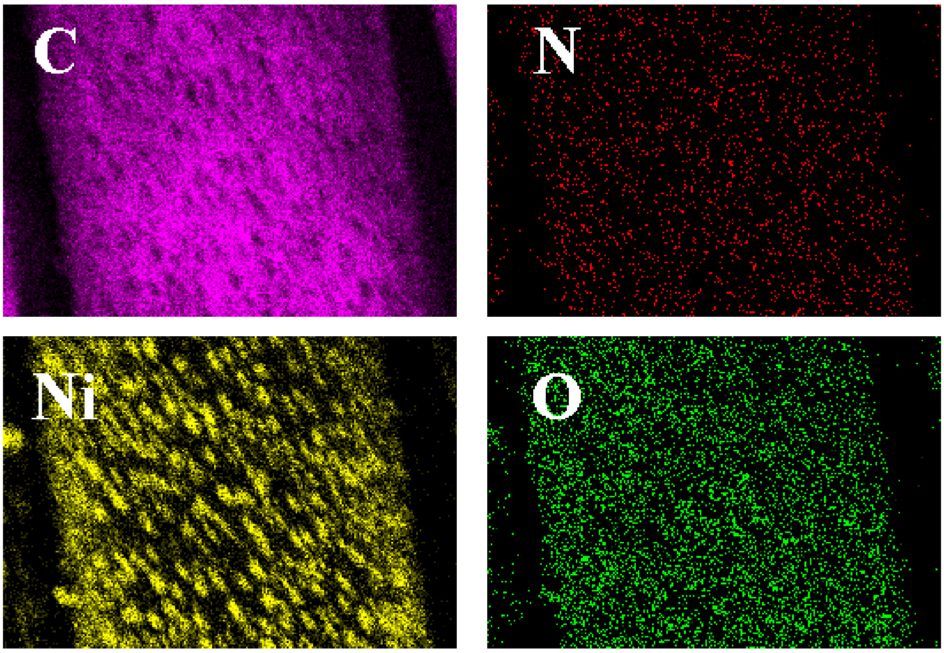

Figure S1. EDS mapping images of NiO@NCNWs-CC.

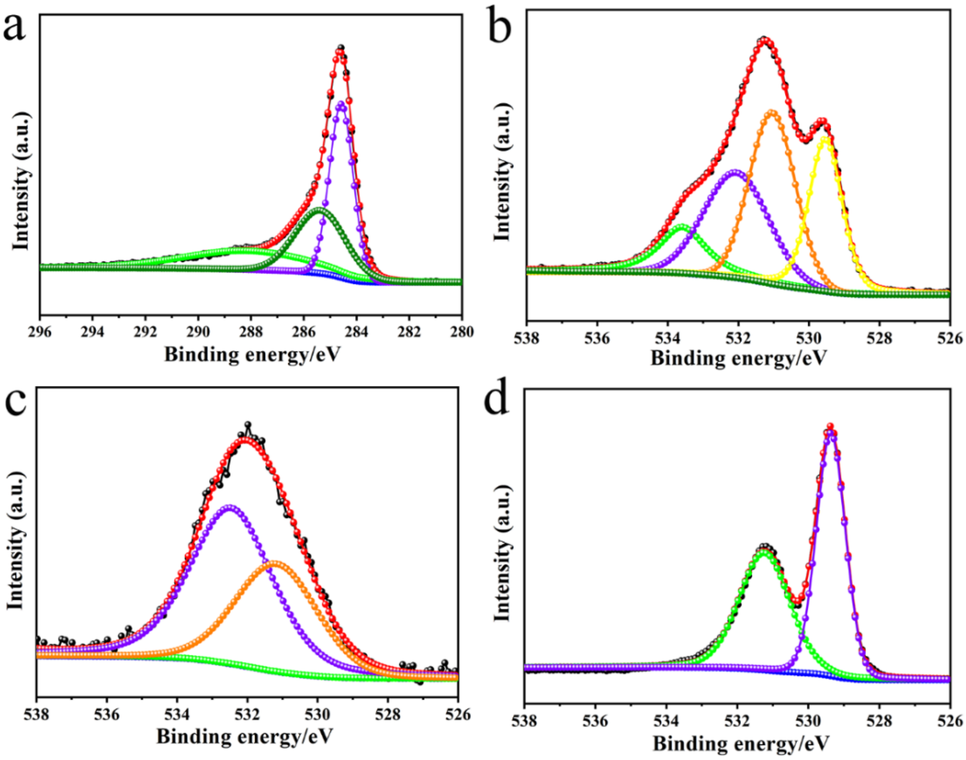

Figure S2. (a) C1s spectrum of NiO@NCNWs-CC; (b-d) O1s spectrum of NiO@NCNWs-CC, NCNWs-CC and NiO-CC.

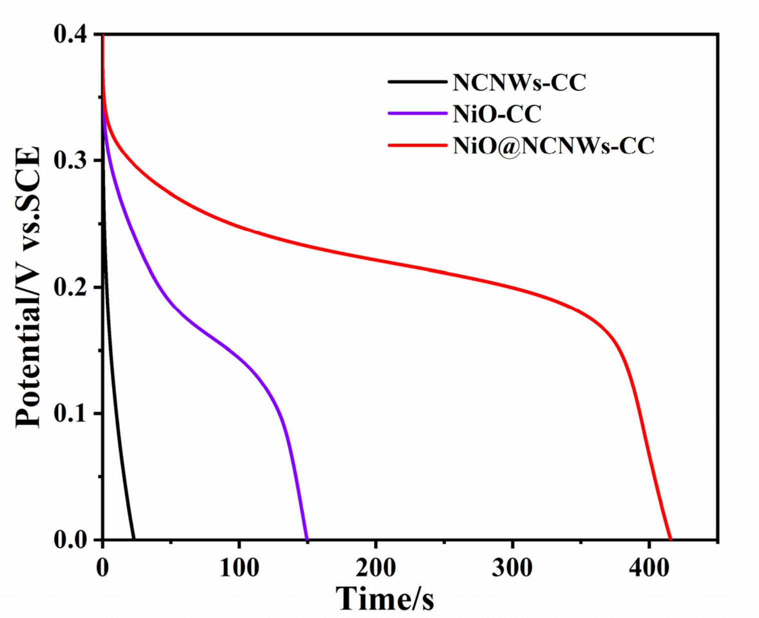

Figure S3. The Galvanostatic discharge curves of NiO@NCNWs-CC, NCNWs-CC and NiO-CC.

Table S1. The specific capacitance of NiO-based electrode materials.

Composition	Morphology	Capacity	Electrolyte	Ref.
**NiO/NCBN/CC**	Nanowires	1039.4 F g^{-1}	6M KOH	This work
**NiO/NC**	hollow	1026 F g^{-1}	6M KOH	1
**Ti_3C_2TX @NiO**	heterostructure hydrogel	966 F g^{-1}	1M KOH	2
**NiO**	nanoparticles	810 F g^{-1}	6M KOH	3
**NiO**	nanoclusters	449 F g^{-1}	6M KOH	4
**NiO**	nanoflakes	410 F g^{-1}	6M KOH	5
**NiO**	nanospheres	982F g^{-1}	6M KOH	6
**NiO**	nanofibers	700F g^{-1}	6M KOH	7
**CO_3O_4/NiO**	nanofilms	710F g^{-1}	6M KOH	8
**NiO@CO_3O_4 @MnO_2**	tremella-like	792.5F g^{-1}	6M KOH	9
**NiO/RGO**	flower-like	900F g^{-1}	6M KOH	10
**NiO/Carbon**	nanospheres	406F g^{-1}	6M KOH	11

References

1. C. Huang, S. Lv, A. Gao, J. Ling, F. Yi, J. Hao, M. Wang, Z. Luo, D. Shu, Boosting the energy density of supercapacitors by designing both hollow NiO nanoparticles/nitrogen-doped carbon cathode and nitrogen-doped carbon anode from the same precursor. *Chem. Eng. J.* 431 (2022) 134083.
2. W. Chen, Y. Peng, Z. Qiu, X. Zhang, H. Xu, 3D hierarchical Ti_3C_2TX@ NiO-reduced graphene oxide heterostructure hydrogel as free-standing electrodes for high performance supercapacitor. *J. Alloy. Compd.* 901 (2022) 163614.
3. S. Chatterjee, R. Maiti, M. Miah, S.K. Saha, D. Chakravorty, NiO nanoparticle synthesis using a triblock copolymer: enhanced magnetization and high specific capacitance of electrodes prepared from the powder, *ACS Omega* 2 (2017) 283–289.
4. P. Pandurangan, T.N. Parvin, B. Soundiraraju, Y. Johnbosco, M. Ramalingam, M. Bhagavathiachari, S.A. Suthanthiraraj, S.S. Narayanan, Ultrasmall NiO nanoclusters modified with conical Ni(II)-SR staples for high performance supercapacitor applications, *New J. Chem.* 41 (2017) 6127–6136.
5. Q. Sun, S. Bao, Effects of reaction temperature on microstructure and advanced pseudocapacitor properties of NiO prepared via simple precipitation method, *Nano-Micro Lett.* 5 (2013) 289–295.
6. F. Yu, L. Zhu, T. You, F. Wang, Z. Wen, Preparation of chestnut-like porous NiO nanospheres as electrodes for supercapacitors, *RSC Adv.* 5 (2015) 96165–96169.
7. M. Zhang, Q. Li, D. Fang, I.A. Ayhan, Y. Zhou, L. Dong, C. Xiong, Q. Wang, NiO hierarchical hollow nanofibers as high-performance supercapacitor electrodes, *RSC Adv.* 5 (2015) 96205–96212.
8. Y. Zuo, J.-J. Ni, J.-M. Song, H.-L. Niu, C.-J. Mao, S.-Y. Zhang, Y.-H. Shen, Synthesis of Co_3O_4/NiO nanofilms and their enhanced electrochemical performance for supercapacitor application, *Appl. Surf. Sci.* 370 (2016) 528–535.
9. H. Wang, Q. Ren, D.J.L. Brett, G. He, R. Wang, J. Key, S. Ji, Double-shelled tremella- like

$NiO@Co_3O_4@MnO_2$ as a high-performance cathode material for alkaline supercapacitors, *J. Power Sources* 343 (2017) 76–82.

10. A. Liu, H. Zhang, G. Wang, J. Zhang, S. Zhang, Sandwich-like NiO/rGO nanoarchitectures for 4 V solid-state asymmetric-supercapacitors with high energy density, *Electrochim. Acta* 283 (2018) 1401–1410.
11. M. Liu, X. Wang, D. Zhu, L. Li, H. Duan, Z. Xu, Z. Wang, L. Gan, Encapsulation of NiO nanoparticles in mesoporous carbon nanospheres for advanced energy storage, *Chem. Eng. J.* 308 (2017) 240–247.
